# Dr. Paris and Mr. Fonblanque on Medical Jurisprudence

**Published:** 1823-12-01

**Authors:** 


*Medical Jurisprudence.* By Dr. Paris and Mr. Fonblanque.

[Second Analytical Article, continued from page 426.]

Our readers will remember that we closed our first analytical article with the subject of Nuisances. The next topic discussed by our authors is headed "Impositions," embracing feigned or simulated diseases, and adulterations of food.

1. *Simulated Diseases.* Various are the motives which have induced man to assume the guise of suffering—as if there was not enough of real affliction on his species. Among these sinister motives are, military exemptions and discharges—civil disqualifications—the hope of parochial relief—shelter of an asylum, or compassion and alms. There are also many instances on record where no other earthly motive could be discovered than the whim or caprice of the individual.

Although an intelligent practitioner will not find much difficulty in detecting these counterfeit diseases, in general, yet he ought to be on his guard not to push his incredulity too far, but to lean to the side of mercy, where there is any doubt on the subject. We have known a man who complained of inability to move the shoulder-joint without much pain, and yet nothing could be seen externally for a month or six weeks, during which time the man was excused from duty. At length the surgeon got tired, and suspected that the man was *sculking*. He was ordered to duty, but came back declaring he could not move his arm. He was reported to the commanding officer as counterfeiting inability, and was actually flogged, though very moderately. It turned out, however, that a deep-seated abscess had been forming in the shoulder-joint, which ultimately terminated in complete anchylosis—and, we need not add to the utter disgrace of the surgeon, whose inhumanity as well as ignorance was a theme among his brother officers for years afterwards.

"Whenever the suspicions of a medical person are excited with respect to the sincerity of a patient's account, he should always endeavour to conceal them; he should become himself a dissembler, *superare malitiam malitia*," for while the impostor is persuaded that the medical attendant is his dupe, he will be less on his guard; he should then be desired to describe with minuteness every symptom and circumstance of his malady; he should be questioned as to its origin, progress, and duration, its seat, and intensity, and the effects produced upon it by remedies; few impostors will be able to withstand such interrogatories without tripping; they will soon betray some incongruity in their statements, and enable the pathologist to elicit the truth. A girl of seventeen counterfeited epilepsy so well in the general hospital of Montpellier, as to elude all suspicion, until *M. de Sauvages*, who, being less credulous, asked her whether she had not felt an air pass from the hand to the shoulder, and from the shoulder to the thigh, when, upon her replying in the affirmative, he ordered her to be whipped, after which she had never any return of the disease." 357.

If a patient complains of long protracted disease which has rendered his life uncomfortable, and yet without any attendant emaciation, we are naturally led to suspect the truth of his statement. We ought, in these cases, to find out whether the patient has, in fact, flown for relief *voluntarily*, to any remedy; "for if he be an impostor, however cheerfully he may have appeared to submit to medical discipline, we shall find, upon minute examination, that he has uniformly neglected every plan proposed for his case." Galen was, from a circumstance of this kind, led to the detection of a person who feigned a fit of cholic, in order to avoid attending a public assembly, but who neglected the remedy (philonium) which had uniformly relieved him, when labouring under actual attacks of the disease, to which he was, in reality, subject.

"When the more ordinary modes of investigation have failed in leading to the detection of an imposture, of whose existence we entertain but little doubt, we may proceed to a system of intimidation, and to severe discipline; few impostors, however sturdy, can withstand the cravings of hunger, blistering, the affusions of cold water, and above all a continual nausea from the administration of divided doses of *Tartarized Antimony;* and yet exceptions of an extrordinary kind might be adduced; 'I have seen an instance,' says *Dr. Hennen*, 'where the patient admitted of all the preparatory measures of amputation before he thought proper to relax his knee joint;' the same author also relates the case of a dragoon who bore very severe riding-school duty for some weeks, secured to his horse, before he could be brought to acknowledge that his chronic rheumatism was assumed. *Mahon* records a very extraordinary instance of a conscript, who feigned blindness, and baffled every attempt to detect the imposition; he was even placed on the margin of a river, and desired to go forward, which he did, and fell into the stream; he was however, without doubt, aware that boats were provided for his safety, for after having received his discharge, he freely acknowledged the imposition which he had practised." 358.

The diseases that have been feigned are very numerous, and the detection of each case will require an exertion of ingenuity, for which no previous instruction can ever provide.

*Insanity* is one of those diseases which have been often feigned, from the earliest antiquity—instance David, Ulysses, and Lucius Brutus. "The feigned lunatic never willingly looks his examiner in the face; and if his eye can be fixed, the changes in his countenance, on being accused, will be strongly indicative of his real state of mind." It is moreover difficult to imitate the habits of a lunatic for any length of time—and especially to forego sleep. An insane person generally sleeps little and talks much during the night, but the pretender, if he thinks he is not watched, will sleep, and act his part only when he supposes he is overlooked.

*Somnolency* has been often assumed, and Dr. Hennen has related a remarkable case in his work on military surgery.

> "A person of the name of *Drake*, in the Royal African Corps, assumed an appearance of total insensibility, under which he resisted every kind of treatment: he resisted the shower bath as well as shocks of electricity; but on a proposal being uttered in his presence to apply the actual cautery, his pulse rose; and on preparations being made to remove him to Bethlem hospital, an amendment soon manifested itself." 360.

We saw this impostor the day he was landed at Portsmouth, and detected the imposture in one minute, and that by a very simple experiment. He was lying on a bench with mouth and eyes closed as if in a profound sleep. We desired a person present to give us a piece of aloes to put into the patient's mouth, first laying a hand on one of his temples. The moment we attempted, with the other hand, to push down the lower jaw, we felt the crotaphyte muscle thrown into action to resist the operation. This convinced us at once of the voluntarity of the act, and we predicted that he would turn out an impostor, which he did. By the way, he kept up the farce till he effected his discharge from His Majesty's service.

A curious case is also related by Dr. Hennen, where violent palpitation of the heart could be produced voluntarily. Dr. H. found that he could, at any time, render the affection very imperfect by throwing the patient's head well back, so as to destroy that voluntary combination of muscular action, on which he believed the palpitation to depend.

*Epilepsy* offers great facilities for the impostor, as it does not require the unremitting caution which other maladies exact for successful imitation. During the feigned attack the blood is generally sucked from the gums, and the mouth made to froth by chewing soap.*

* See evidences before a committee of the House of Commons on the subject of mendicity.

" There is, however, one symptom of the disease which cannot be imitated—the incontractility of the pupil of the eye, on exposure to light, which in a real fit of epilepsy is always dilated and immoveable; nor is the patient affected by rubbing stimulants on the nose." 361.

We know from actual observation that the abovementioned state of the pupil is by no means invariably present in real epilepsy, because we have seen it otherwise in private patients, where no possible reason could exist for feigning the malady, and where they were extremely anxious to get cured.

In respect to hysteria, it is almost impossible to detect a well counterfeited paroxysm. Cullen was deceived by a man who simulated this disease as long as suited his convenience in the Royal Infirmary of Edinburgh, and afterwards had a hearty laugh at the doctor for his credulity.

It may be remarked, that these spasmodic affections sometimes assume an extravagance of character and action that might render them suspicious, were they not authenticated beyond all doubt. The jumpers in Cornwall are an instance in point; and shew the powerful influence of sympathy in propagating a spasmodic paroxysm. It is pretty well known that epileptic seizures have sometimes been communicated to certain bye-standers of susceptible dispositions.

" Dum spectant oculi læsos, læduntur et ipsi."

It is properly observed by our authors that, in such cases, although we must consider those in whom the affection originated as designing impostors, we are bound to acquit the general mass of sufferers of any blame, except that which may attach to excessive credulity. We pass over several pages of simulated diseases as not containing any thing worthy of notice, concluding in the words of our authors.

" We shall now dismiss the subject of simulated diseases, leaving such deceptions as that of *Miss M'Avoy* of Liverpool, to the fate which must await them; and the professional men who have aided them by their credulity, to the contempt which they so richly merit, from the more enlightened part of their medical brethren." 373.

2. *Adulteration of Food.* Mr. Accum's " Death in the Pot," though greatly exaggerated, no doubt, is acknowledged to contain many truths. It cannot, indeed, be denied, that a great part of our daily food, and a still greater portion of our luxuries are the constant objects of fraudulent adulteration. Nor can we hope for amendment while the temptations remain so excessive, the detection so difficult, and the punishment so inadequate to the crime—above all, while the trouble and expense of prosecution continue to be so disproportioned to the injury sustained by any individual.

The adulteration of bread by alum has excited much alarm among the community; but, for our own parts, we cannot believe that the quantity (ten or fifteen grains in the quartern loaf) can have any prejudicial influence on the most delicate constitution. The adulteration by gypsum, pipe-clay, and chalk, is a much more serious evil. It is admitted that there is no unexceptionable test for the detection of alum in bread, since there are impurities in the salt used by the bakers sufficient to throw down precipitates like those we might attribute to alum.

In respect to beer and porter, they have the name of being strongly adulterated—perhaps not so much in the cauldrons of the brewers as in the barrels of the publican.

"The following are the more usual additions made by the publican; *beer-heading*, which is intended to impart the "*cauliflower head*," and consists of sulphate of iron, common salt, and alum, for which several convictions have taken place, (*Minutes of the Committee, above cited;*) it is necessary to observe that the addition of this "*heading*" is made with a view to restore the property of frothing to the porter, which has been destroyed by dilution with table beer. The extract of the berries of the *Coculus Indicus*, possessing properties eminently narcotic, is added for a purpose too obvious to require explanation, and is regularly sold by the brewer's druggists under the technical appellation of "BLACK EXTRACT." There is also another preparation, for a similar object, sold under the name of "BITTERN," and which is a compound of *black extract*, *extract of quassia*, *spanish liquorice*, and calcined *sulphate of iron*. "MULTUM," used as a substitute for malt and hops, consists of *Extract of Quassia*, and *Liquorice*. We must close this note by expressing our regret at the little assistance to be derived from chemistry in the detection of such frauds; mineral substances, as *sulphate of iron*, or any of the mineral *acids*, can certainly be recognized in our laboratories; but when we attempt to identify vegetable principles, the resources of analysis completely fail." 378.

Milk is rarely adulterated with any other ingredient than the crystal spring—if that can be called adulteration. The subject is no farther pursued by our author.

3. *Policies of Insurance on Lives.* Death, by suicide, or the hands of justice, is generally excepted in all policies. And if there be any fraudulent concealment as to the state of the party's health or age, the policy is void.

"But 'even where there is an express warranty that the person is in good health, it is sufficient that he is in a reasonable good state of health; for it never can mean that the *cetui que vie* is perfectly free from the seeds of disorder. Nay, even if the person, whose life was insured, laboured under a particular infirmity, if it can be proved by medical men, that it did not at all, in their judgment, contribute to his death, the warranty of health has been fully complied with, and the insurer is liable.'' 383.

Survivorship winds up the second part of the work before us, and from this we can extract nothing but *law*, of which we profess ourselves to be entirely ignorant.

We are now arrived at the most important division of the work, comprehending the principal pleas of the crown, three of which, rape, arson, and murder, are pre-eminently the subjects of medical jurisprudence.

1. *Arson*. This crime, at common law, is the malicious and voluntary burning of the house of another, by night or day. The charge may occasionally become the subject of scientific research, and the accused individual receive an honorable acquittal at the hands of the chemical philosopher, by whose interposition the conflagration, unjustly imputed to malice, may be proved to have originated from a spontaneous process of decomposition.

Spontaneous combustion (an inflammation occasioned by the reaction of different bodies upon each other, at the ordinary heat of the atmosphere, without the contact or approach of any other body previously raised to a high temperature) has attracted the attention of many eminent chemists, and numerous experiments have been instituted to throw light upon the causes of this curious phenomenon. The following may be considered as the principal sources from which it may originate.

"I. Friction.

"II. Fermentation of Vegetable and Animal Substances, as that of *hay*, *oatmeal*, *roasted bran*, *coffee*, *&c. rags in paper-mills*, *&c.*

"III. Chemical Action. *Accension of oils, by various animal, vegetable, and mineral substances; accension of vegetable matter by concentrated acids; ignition of lime by the affusion of water; ignition of pyrites.*" 403.

*Friction.* Two pieces of wood rubbed hard together will bring forth fire—and all the chemical philosophers of Europe cannot explain this simple phenomenon. "We must therefore rest upon it as an ultimate fact, and be satisfied with availing ourselves of the advantages to which a knowledge of it may conduce."

*Fermentation.* The presence of water seems indispensible here—hence in all cases of spontaneous combustion from fermentation, the substances have either been imbued with moisture, or have possessed the power of absorbing it from the atmosphere.

"The firing of hay, when stacked in too moist a condition, is a striking exemplification of this fact; the same circumstance occurs from great accumulations of turf, flax, and hemp, heaps of linen rags in paper-mills, &c. provided a sufficient portion of moisture be present to excite the process of fermentation, and the consequent evolution of heat. *Oatmeal*, from the extreme avidity with which it imbibes water, and the heat which is generated by the absorption of it, is necessarily liable to spontaneous combustion; the following well authenticated case may serve as an illustration of this fact: 'A gentleman removed with his family from Glasgow to Largs, in May last, and shut up his house, which was not re-opened until the end of August; the house stands on the side of a deep declivity, so that the kitchen which is in the back part, though sunk considerably below the level of the street, is entirely above ground, and is well lighted and ventilated. In an opening of the wall, near the kitchen fire-place, originally intended it is supposed for an oven, there was placed a wooden barrel bound with iron hoops, and filled with *oatmeal*. This meal, which had heated during the absence of the family, at last caught fire, and was totally consumed, together with the barrel which contained it, nothing remaining but the iron hoops and a few pieces of charcoal." 405.

In some cases torrefaction increases the propensity of vegetable substances to spontaneous combustion, as coffee, roasted French beans, lentils, &c. Some years ago a fire broke out at Nauslitz, said to have been occasioned by the application of roasted bran to the necks of some cattle in a wooden cow-house. M. Rude instituted some experiments, by which he found that if rye bran, roasted until it acquires the colour of coffee, be wrapped up in a linen cloth, it will shortly take fire. Montet relates that animal substances will sometimes take fire, and says, he himself witnessed the spontaneous accension of a dunghill. Woollen stuffs are said to have taken fire spontaneously.

*Chemical Action*. This is a frequent cause of combustion. Fixed oil, especially when of a drying nature, with its various accomplices from the animal, vegetable, and mineral kingdom, has, in darkness and secresy consigned ships, houses, and manufactories to the flames.

"About twenty-five pieces of cloth, each of which contained nearly thirty ells, were deposited upon wooden planks in a cellar at Lyons, on the eighth of July, 1815, in order to conceal them from the armies which then over-ran France; *in the manufacture of the cloth 25lbs of oil were used for a quintal of wool, and the cloth was quite greasy*, each piece weighing from 80lbs to 90lbs; the cellar had an opening to the north, which was carefully shut up with dung, and the door was concealed by bundles of vine-props, which freely admitted the air; on the morning of the 4th of August an intolerable stench was perceived, and the person who entered the cellar was surrounded by a thick smoke, which he could not support; a short time afterwards he re-entered with precaution, holding a stable lanthorn in his hand, and he was astonished to perceive a shapeless glutinous mass, apparently in a state of putrefaction; he then removed the dung from the openings, and as soon as a circulation of air was established, the cloth instantly took fire. In another corner of the cellar lay a heap of stuffs which had been *ungreased* and prepared for the fuller, *but they had suffered no change.* In this case the agency of the oil was sufficiently evident. In June, 1781, a similar occurrence happened at a wool-comber's in a manufacturing town in Germany, where a heap of wool-combings, piled up in a close warehouse seldom aired, took fire spontaneously; this wool had been, by little and little, brought into the warehouse, and from want of room, been piled up very high and trodden down; that this combed wool, to which rape oil mixed with butter had been added in the combing, burnt of itself, was sworn to by many witnesses; one of whom affirmed that ten years preceding a similar fire had happened among the flocks of wool at a clothiers, who had put them into a cask, where they were rammed down hard for facility of carriage, and that this wool burnt from within outwards, and became quite a cinder. Cotton goods, in which linseed oil had been spilt, have burnt in a similar manner, and there is reason to attribute to an accident of this kind the recent loss of a merchant-vessel homeward bound from the East Indies."* 407.

Amongst the mineral substances capable of exciting the inflammation of oils, an ore of manganese, called the *black wad of Derbyshire*, is remarkable. When this substance is powdered and mixed with a little linseed oil, it will ignite in the course of an hour, and become red hot, like burning small-coal. It is supposed that the Pantheon in Oxford-

---

* We have no doubt that many of those vessels which are annually consumed by fire in the harbours of Bombay, the Ganges, and other oriental ports, are lost in this way.

We well remember an accident of this kind, that nearly proved fatal to ourselves about eighteen years ago. The writer of this note was a passenger in the Russel of 74 guns, on a voyage from Malacca to Madras, in the year 1805. In the middle of a dark and stormy night, a fire broke out in the purser's store-room, (occasioned by some oil getting loose among cotton yarn kept for lamp-wicks) close to the powder magazine. Fortunately the officer of the watch, without sounding any alarm, took about a dozen of steady men with him, each carrying a bucket of water, and quenched the flame before it communicated with the magazine. A fellow-passenger who happened to be on deck when this fearful intelligence was reported, ran down to the ward room where the writer was lying in a cot, but finding him fast asleep he had the humanity not to awake him, expecting that the ship would blow up in a few minutes!

street was destroyed by the combustion of a composition of this kind used for painting the scenery. A fire lately took place in Mr. Parke's chemical laboratory in consequence of the leakage from a carboy of nitric acid. It is well known that the sudden slacking of quick-lime has occasioned fires. At Edmonton, last winter, the flood, consequent on a heavy fall of rain, made its way among the quick-lime in a brick-layer's premises, which took fire and were burnt.

"There still remains for notice another source of spontaneous burning,—the ignition of *Pyrites,* and that of cinders from the furnaces of glass-works, from exposure to air and moisture; it was in this manner that the ship *Ajax* was supposed to have been consumed, from the spontaneous combustion of coal, abounding in *Pyrites.*

*Human Combustion.* Our authors remark that the combustion of human beings has been *erroneously* designated as *spontaneous,* "for in every recorded instance the approach of some burning body, as that of the flame of a candle, or an ignited pipe, appears to have been necessary for its occurrence."

"It can no longer be doubted,' says *Dr. Gordon Smith,* 'that persons have retired to their chambers in the usual manner, and in place of the individual, a few cinders, and perhaps part of his bones, were found.' Upon this occasion we confess ourselves more sceptical; the phenomenon is contrary to all our preconceived views, and must therefore require more than ordinary testimony for its support, although we are ready to admit, that upon any other less miraculous subject, evidence even less powerful than that produced on the present occasion, would be deemed amply sufficient." 412.

Now we would reason thus:—We have no more à priori or *preconceived* cause for believing or doubting the spontaneous combustion of the materials composing the human frame, than of any other substance, simple or compound, in the material world around us. The whole must be determined by the evidence of facts, and not by "preconceived views." Plouquet enumerates 28 cases. Dr. Trotter adduces a considerable number of instances of persons addicted to drunkenness having undergone spontaneous combustion; and the journals of various nations present us with a great variety of examples—"all of which, say our authors, with some slight shades of difference, appear to have been attended with the same phenomena:—a fact which we freely admit affords internal evidence of their authenticity." On the other hand, they observe, that amidst all these cases *only one* is related where the person survived, for a short time, and gave an account of the manner in which "he was *struck* with the fire."

"This was the case of the priest *Bertholi*, described in one of the Journals of Florence for October 1776, by *M. Battaglia*, the surgeon, who attended him; we extract a short account of this extraordinary event from *Foderé* (tom. 8, p. 210) who to his own observations on the subject adds those of *Fouquet*, *Marc*, *Koop*, and others. *Don Gio Maria Bertholi*, having spent the day in travelling about the country, arrived in the evening at the house of his brother-in-law; he immediately requested to be shewn to his destined apartment, where he had a handkerchief placed between his shirt and shoulders, and being left alone, betook himself to his devotions. A few minutes had scarcely elapsed when an extraordinary noise was heard from the apartment, and the cries of the unfortunate priest were particularly distinguished; the people of the house hastily entering the room, found him extended on the floor, and surrounded by a light flame which receded (*à mèsure*) as they approached, and finally vanished. On the following morning, the patient was examined by *M. Battaglia*, who found the integuments of the right arm almost entirely detached and pendant from the flesh; from the shoulders to the thighs the integuments were equally injured; and on the right hand, the part most injured, mortification had already commenced, which notwithstanding immediate scarification rapidly extended itself. The patient complained of burning thirst, and was horribly convulsed, he passed by stool putrid and bilious matter, and was exhausted by continual vomiting accompanied by fever and delirium. On the fourth day, after two hours of comatose insensibility, he expired; during the whole period of his suffering, it was impossible to trace any symptomatic affection. A short time previous to his decease, *M. Battaglia* observed, with astonishment, that putrefaction had made so much progress that the body already exhaled an insufferable odour, worms crawled from it on the bed, and the nails had become detached from the left hand.

"The account given by the unhappy patient was, that he felt a stroke like the blow of a cudgel on the right hand, and at the same time he saw a lambent flame (*bluette de feu*) attach itself to his shirt, which was immediately reduced to ashes, his wristbands (*poignets*) at the same time being utterly untouched. The handkerchief, which, as beforementioned, was placed between his shoulders and his shirt, was entire, and free from any trace of burning; his breeches were equally uninjured; but though not a hair of his head was burnt, his coif (*calotte*) was totally consumed. The weather on the night of the accident was calm, the air very pure; no empyreumatic or bituminous odour was perceived in the room, which was also free from smoke; there was no vestige of fire, except that the lamp, which had been full of oil, was found dry, and the wick reduced to cinder." 415.

The following are the circumstances in which all the recorded cases so singularly concur.

"1. The persons who have suffered this species of combustion have been long accustomed to drink spirituous liquors.

"2. These persons have been generally females, and advanced in years.

"3. The body has not burned *spontaneously*, but accidentally, in as much as it required for its inflammation the contact or approach of some burning body, or that of electric matter.

"4. The extremities of the body, such as the feet and hands, have in general escaped.

"5. The fire has little injured, and sometimes not at all, those combustible things that were in contact with the body when it was burning.

"6. The combustion of these bodies has left a residue of greasy and fœtid ashes and fat, that were unctuous, and extremely offensive and penetrating." 415.

It is needless to advert to the various theories which have been proposed for the explanation of this singular phenomenon. Our authors justly observe that, if the bodies in question were actually found consumed in the manner described, it is quite impossible that they were burnt by ordinary means; for even admitting that they had been rubbed over with highly combustible substances, the explanation would not be less difficult, since it is well known that when criminals were condemned to death by fire, a very large quantity of combustible materials was required for burning their bodies.

2. *Rape.* This, which is the violent deflowering of a female against her will, is felony by the common and statute law. It is also lawful for the female to take the life of the ravisher, or for the husband or parent of the female, to put the culprit to instant death. Formerly it was necessary that the injured female should make immediate complaint; but now no time is fixed. A jury, however, will seldom listen to a stale complaint. In a medical point of view it would be highly desirable that immediate inspection should be made, as many collateral proofs would be effaced in a few hours.*

---

"* The injuries thus occasioned, consist in rupture of the hymen, swelling, contusion, inflammation, or laceration of the parts, discharge of blood; and in persons of extreme youth, the laceration of the perineum is said to have sometimes occurred; and as Rape cannot be completed without considerable violence, we should also expect to find marks of force in other parts of the body, such as bruises about the arms and thighs; but in appreciating the value of such indications, let the practitioner remember, that the greater part of them may occur where the connexion has taken place with the consent of the female, or they may even be the effect of disease. Dr. *Percival* relates a case where the inflammation of the pudenda, and symptoms of defloration occurred in a child of four years old, which occasioned her death; there

As the medical witness will most likely be among the first who sees the parties accused and accusing, he should certainly have his wits about him, as he will be called upon by the Court for general as well as professional evidence.

A female child under twelve years of age, is, in law, deemed incapable of consenting to any act, much less to her dishonour. The carnal knowledge therefore of such an infant is virtually a rape. We find so little that concerns the medical evidence on this disgusting subject in the work before us, that we shall pass on to the succeeding section.

3. *Homicide.* It is to aid the administration of justice, in cases of homicide, that the medical practitioner is most frequently called upon to give professional evidence. It is therefore necessary that he should, in the first place, determine by minute examination, inspection, or dissection, whether death was caused by violence, or attributable to natural causes. The subject divides itself into several branches, and these we must follow seriatim.

*Real and Apparent Death.* The difference between these two states is not so easily defined as one would expect.

Putrefaction, considered by many authors as unequivocal evidence, does not display itself immediately after death. On the other hand, in certain morbid states of the living frame, so feebly do the powers of life resist the operation of physical agents, that a consideration very analogous to putrefaction obtains before respiration and circulation cease. We lately saw a remarkable instance of this kind, in the person of an elderly man of the name of Hill, in Great Windmill-Street. He had been for a considerable time affected with hydrothorax, and at length sphacelus of the integuments of the lower extremities took place, and spreading from thence almost the whole surface of the body became black and sphacelated before he expired. It is properly remarked by our authors, that the test of death must rather be sought for amongst those signs which indicate the quiescence or cessation of the functions of life, than from those which manifest the decomposition of the organs by which these functions are performed.

---

were strong reasons for suspecting that she had been injured by a boy of fourteen years of age, and he was accordingly taken into custody; but the case received elucidation from several others of a similar nature having been shortly afterwards received into the same hospital, and of whose nature no doubt could be entertained." 418.

"The total cessation of respiration, pulsation, sensation, and all motion, it might be supposed, would indicate, to the least experienced, the departure of life, while the general aspect of the body, its pale and livid hue, the coldness of its surface, and the stiffness of its limbs, we might conclude were signs so palpable and satisfactory as to defy the possibility of doubt. To the skilful medical practitioner we apprehend such signs must ever be unequivocal; but we are not prepared to say that a common observer may not be sometimes deceived by them; in cases of extreme debility, as in the latter stage of fever, and where the patient is confined in vitiated air, the exhaustion may be so considerable as to lend all the appearance of death; indeed, that such cases have occurred we have no less a testimony than that of the philanthropic *Howard*, who, in his work on Prisons, says, 'I have known instances where persons supposed to be dead of the gaol fever, and brought out for burial, on being washed with cold water, have shewn signs of life, and soon afterwards recovered.'" Vol. ii. p. 4.

We do not deem it necessary, however, to amuse, or rather tire the reader with narratives of those numerous *nugæ canoræ* that fill the pages of some popular productions on the subject of trances, premature interments, and extra-ordinary resuscitations. The public always have, and always will betray a morbid curiosity for such stories, whether true or false—but, for our own parts, we are very sceptical on these subjects.* We cannot, therefore, compliment the poetical erudition except at the expense of the judgment of our authors, in quoting scenes from Shakspeare or fables from Tasso, in a work on Medical Jurisprudence. Certes a court of law or a coroner's jury will not lend much ear even to "the sighs of Clorinda issuing with her blood from the trunk of the cypress;" nor to "the sufferings which attended the confinement of Ariel, by the witch Sycorax within the rift of a cloven pine."

———" 'She did confine thee,
By help of her more potent ministers,
And in her most unmitigable rage,
Into a cloven pine; within which rift
Imprison'd, thou didst painfully remain
A dozen years.'"

———" 'Thou best knows't
What torment I did find thee in: thy groans
Did make wolves howl, and penetrate the breasts
Of ever-angry bears; it was a torment
To lay upon the damn'd.'"

"*Tempest, Act* 1, *s.* 2."

* See our review of Mr. Whiter in a former number of the Journal.

We should hold ourselves not justifiable in quoting these passages from the original authors; but we have sufficient excuse in bringing them forward for the support of our general charge of irrelevancy and supererogation in a didactic work on a grave subject. The following passage, though not free from the besetting sins of the whole publication, contains enough of redeeming matter to merit a place in our pages.

"We feel no hesitation in asserting that it is physiologically impossible for a human being to remain more than a few minutes in such a state of asphyxia, as not to betray some sign by which a medical observer can at once recognise the existence of vitality, for if the respiration be only suspended for a short interval, we may conclude that life has fled for ever; of all the acts of animal life this is by far the most essential and indispensible; *breath* and *life* are very properly considered in the Scriptures as convertible terms, and the same synonym, as far as we know, prevails in every language. However slow and feeble respiration may become by disease, yet it must always be perceptible, provided the naked breast and belly be exposed; for when the intercostal muscles act, the ribs are elevated, and the sternum is pushed forward; when the diaphragm acts, the abdomen swells; now this can never escape the attentive eye, and by looking at the chest and belly we shall form a safer conclusion than by the popular methods which have been usually adopted, such as the placing a vessel of water on the thorax, in order to judge by the stillness or agitation of the fluid; or holding the surface of a mirror before the mouth, which, by condensing the aqueous vapour of the breath, is supposed to denote the existence of respiration, although too feeble to be recognised in any other way.

————" 'Lend me a looking-glass;
If that her breath will mist or stain the stone,
Why, then she lives.' " " *Lear*, *Act* v. *s*. iii."

"For the same purpose, light down, or any flocculent substance, from the extreme facility with which it is moved, has been supposed capable of furnishing a similar indication; but the result must not be received as an unequivocal proof, and accordingly *Shakspeare*, with that knowledge and judgment which so pre-eminently distinguish him, has represented *Prince Henry* as having been thus deluded, when he carried off the crown from the pillow of *Henry* the *Fourth*.—

————" 'By his gates of breath
There lies a downy feather, which stirs not.
Did he suspire, that light and weightless down
Perchance must move.' " 12.

It may be remarked, however, that an imperceptible current of air may agitate the light down, and thus simulate the effects of respiration ;—while an exhalation, totally unconnected with that function, may sully the surface of a mirror held before the mouth.

In respect to the circulation, the absence of pulsation in the heart and arteries is no infallible criterion of death. In many people the action of the heart is not to be felt in health; and we have seen instances of non-pulsation in any tangible artery for hours together. Stiffness of the body is another criterion which has been much insisted on. It is not unequivocal; but as a medical practitioner can hardly be deceived, after he has examined all the functions and circumstances, we shall pursue the subject no farther.

*Physiological Causes and Phenomena of Sudden Death.*—The aphorism of Bichat holds true, with very few exceptions, that, when death takes place suddenly, it must be from the cessation of the functions of the heart, brain, or lungs; although it is difficult to say which of these organs is the first to fail in its action. The exceptions are cases of sudden death in which the principle of animation would seem to be at once annihilated in every part of the machine—every organ appearing simultaneously affected, as from the agency of intense cold, lightning, electricity, &c.

We shall not dwell on the various hypotheses that have been invented to explain the mysterious connexion and dependence existing between the functions of the heart and lungs. We know that when respiration ceases the action of the heart soon ceases also. But we are very far from being so completely satisfied with the explanation of Bichat as our authors appear to be.

> "It was reserved for *Bichat* to offer a true explanation of this phenomenon: he has very justly stated that, in consequence of the suspension of the respiratory function, the coronary vessels, by which the muscular structure of the heart is supplied, are compelled to carry black, instead of scarlet blood; a fact which in itself is quite adequate to explain the cause of the heart ceasing to contract; for the irritability of this, like that of every other muscle can be alone maintained by duly oxygenized blood." 20.

We imagine there is much to be learnt respecting the physiology of the circulation in all its bearings, and especially in the above respect. M. Lallemand has published the history of a fœtus, in which the brain and spinal marrow were wanting, and yet it even exceeded the usual size. It died, of course, when it was separated from the mother, because the diaphragm and other muscles of respiration were unable to perform their functions without the aid of nervous excitement. We lately examined the heart of a child seven or eight months old, (a patient of Mr. Delph's of Edmonton,) where there was no pulmonary artery. A free communication existed between the auricles and ventricles of the heart, so that, in fact, it was only a single heart; for the blood brought from the general circulation by the cavæ was passed from the right to the left side of the heart, and from thence into the aorta. The pulmonary veins could scarcely be distinguished, and the lungs could only have received blood by the bronchial arteries. The child, however, lived seven or eight months, subject to fits and several anomalous affections, by which it was cut off at last. It is curious that there was little if any blue colour, except during the paroxysms. By these observations we do not deny that cessation of pulmonary function causes cessation of the heart's action. We only demur to the explanation given by Bichat. Our own opinion is, that when respiration ceases the circulation through the lungs soon ceases, and then the heart is no longer supplied with blood of any kind—consequently its function also soon ceases. On the other hand, when artificial respiration commences, the circulation through the lungs commences, or is accelerated, and supplies the heart with blood. The circulation of the fœtus, where the lungs are collapsed, is no objection, because the foramen ovale being open, the collapsed lung offers no impediment to the circulation. After breathing has once commenced, however, the case is altered. The foramen ovale closes, and cessation of respiration produces, we think, cessation of circulation through that organ. By a quotation farther on it will be seen that when respiration ceases the right ventricle is found distended, and the left empty—shewing that the passage of blood through the lungs very soon ceases when respiration ceases, and thus gorges one side of the heart, while the other empties itself into the aortic system.

In respect to the connexion between the brain (with its appendages) and the heart and lungs, it is considered as proved "that the brain is immediately necessary to life, *only* because the muscles of respiration owe their action to its influence."

"A question has arisen, says *Mr. Brodie, (Manuscript Notes)* whether the whole of the brain is essential to the function of respiration, or whether the power of calling the respiratory muscles into action may not reside in some particular part of that organ? It has been stated by *Le Gallois* that if you expose the cavity of the cranium, and remove the upper part of the brain, the muscles of respiration continue to act as usual; if, however, the dissection be continued, as soon as that portion of the *Medulla Oblongata* is removed which corresponds to the *Corpora Olivaria*, their action is immediately suspended. The theory which such an experiment naturally establishes has received no inconsiderable support from the history of a fœtus, published by *Mr. Lawrence* in the Medico-Chirurgical Transactions: in this monster the *cerebrum* and *cerebellum* were entirely absent, but the *Medulla Spinalis* was continued for about an inch above the *Foramen Magnum* of the occiput, so as to form an imperfect *Medulla Oblongata*, and to give origin to several nerves. Death did not take place immediately after birth, as in other instances of cerebral deficiency, but the child breathed for four days after it had been expelled from the uterus." 20.

"In farthur illustration of these views, let us observe the mode in which death takes place in apoplexy, or in cases of pressure on the brain, whether occasioned by a depressed portion of bone, or by blood extravasated within the cranium. At first the patient is insensible to all external impressions, but the breathing is not affected; after an interval, however, the respiration becomes difficult and laborious, and the purple hue of the lips and cheeks, from the subcutaneous vsesels, demonstrates that the blood is imperfectly oxygenized. *The arterial action becomes more slow, in proportion only as the respiration is more difficult; and the pulse may even be distinguished at the wrist, after the breathing has altogether ceased;* under such circumstances it is obvious that life might be protracted for several hours by artificial inflation of the lungs, but as no ultimate benefit could be derived from such an operation, its expediency may be fairly questioned." 22.

Upon the passage marked in italics in the above quotation, we may remark that we have seen many people die of apoplexy, but not one exhibited the phenomena of pulse at the wrist after respiration had altogether ceased. On the contrary, (and we appeal to the personal experience of every individual,) the pulse disappears, in nine cases out of ten, before the respiration ceases entirely. Neither does our experience correspond with that of our authors respecting the pulse, becoming *more slow* in proportion to the difficulty of breathing. In the great majority of cases that we have seen, it was just the reverse. It became quicker and more irregular as the final catastrophe advanced. If this be doubted, let our authors turn to Dr. Cooke, and hear what he says. "It is," says he, "at first regular, strong, full, *slow*, beating from 55 to 65 times in a minute; but as the disease advances, it becomes weaker and *more frequent;* and in the end irregular and intermittent." Upon the whole, we consider the above passage as one that is warped by theory, and made to bend in accordance with a physiological problem, rather than the offspring of unbiassed observation at the bedside.

*Syncope.* From this section we cannot glean any thing interesting. The following aphorism appears to contain the marrow of the whole. "It would appear that where the heart has ceased to pulsate, *in consequence of the cessation of respiration, it can never again be set in motion;* but that where it has stopped from other causes, as from the operation of certain poisons, its muscular irritability not having been exhausted, its action may be occasionally revived." 26.

*Suffocation*—defined the destruction of life from suspended respiration occasioned by *external violence* without this last adjunct, the definition would be too extensive, embracing apoplexy, fatal intoxication, and various diseases of the brain and spinal marrow. It has been shewn by Bichat, our authors observe, that when dark coloured blood is injected into the vessels of the brain, the functions of that organ become immediately disturbed, and soon cease.

"It is not until *after* the full effects of the suspended respiration are thus produced on the *brain*, that the motions of the *heart* become enfeebled, and that the ventricles contract less powerfully, and at longer intervals; at length, the action of the heart is altogether arrested, and if the thorax be examined at the instant that the circulation has ceased, nothing is observed, except a slight tremulous motion of the auricles; *the cavities of the left side are much contracted, and contain only a small quantity of blood, while the right auricle and ventricle, and the large vessels communicating with them, are distended to an unusual size.*" 33.

Now how does this passage accord with the physiology of our authors, which makes the cessation of the heart's action depend on black blood in the coronary arteries? In the above passage it appears that this cessation does not take place till after the full effects of unoxigenated blood are produced on the *brain*. Again, if it be black blood in the coronary arteries which stops the heart's action, how is it that one ventricle is fouud full and the other empty on opening the chest? Does the coronary artery supplying the left ventricle carry blood of a redder colour than that supplying the left, to enable it to act after the other has ceased? Does not this passage plainly prove that in a few minutes after respiration ceases, blood ceases to pass through the capillary system of the lungs, in consequence of which the right ventricle becomes distended, while the left contracts on till no blood is left for it to act upon?

*Drowning.* It is hardly necessary to remark on the antiquated and erroneous physiology which attributed death in drowning to water getting into the lungs instead of air. An animal might as well attempt to breathe fire as water—the muscles of the glottis refusing access to any but the natural element of the lungs—air. We shall give a quotation exhibiting the physiology of drowning as far as our knowledge at present extends.

"If a small animal be immersed in water, contained in a transparent glass vessel, the phenomena of drowning are readily discernible; there is first a deep expiration, by which bubbles of air are expelled from the lungs; there is then an effort to inspire, but the effort is ineffectual; there being no air which can be received into the lungs, and a spasm of the muscles of the glottis seems to forbid the admission of any considerable quantity of water into the trachea. The attempts to breathe are repeated several times, and at each attempt at expiration a small proportion of air is expelled from the mouth and nostrils, until the air-cells of the lungs are almost emptied;* then the animal becomes insensible; and convulsive action of the voluntary muscles mark the instant when the brain begins to suffer from the influx of the dark coloured venous blood. After the cessation of these convulsive actions, the animal becomes motionless, and gives no sign of life; but if the hand be applied to the thorax, the actions of the heart, gradually becoming fainter and fainter, indicate that some remains of vitality still linger in the system. Before the circulation of the blood altogether ceases, the muscles of respiration once more resume their actions, and ineffectual efforts are made to breathe. It is a remarkable circumstance that the diaphragm continues to exert itself nearly as long as the heart itself, and that the interval between the cessation of the motions of the diaphragm and that of the motions of the heart, which is so short in animals that die by strangulation,

* "*Mr. Coleman* examined the lungs of a cat which had been drowned, by placing a ligature on the trachea, removing the lungs from the thorax, and then making an opening in the trachea under water, so as to collect the air which issued from the orifice; the whole quantity of air thus obtained, amounted only to half a drachm; yet the same lungs when inflated, required as much as two ounces of air, by measure, for their distention. Nor would the presence of water appear to be immediately fatal, when introduced into the lungs; *Dr. Goodwin* poured two ounces of water into the lungs of a cat, through an opening made between the cartilages of the trachea; the animal had an immediate difficulty of breathing, and a feeble pulse, but lived several hours afterwards without much apparent inconvenience; it was at length strangled, and the water was found in the lungs. From which it would appear, that the admission of a certain portion of water, does not tend to hasten death. The author of this note was present at an experiment made by *Mr. Brodie*, in which he drowned a guinea pig, whose trachea had been previously perforated; so that in this case, no spasm of the glottis could arrest the ingress of the water into the pulmonary air cells; but this produced no modification of the usual symptoms; nor did it prevent the resuscitation of the animal, which was afterwards effected by the appropriate methods."

is still shorter in those who perish by drowning. These phenomena follow each other in rapid succession, and the whole scene is closed, and the living animal is converted into a lifeless corpse, incapable of recovery, in the brief space of a few moments. *(Brodie's Manuscript Notes.)* If however the animal be taken out of the water before the total extinction of life, and the diaphragm contract afterwards, so as to draw air into the lungs before the action of the heart has ceased, the circulation is maintained, and the animal continues to respire; he will thus have escaped immediate death from suffocation; but his life still remains in jeopardy, for there is a second period of danger, and one at which death may take place, when we are the least prepared to expect it; for the dark coloured blood which has been transmitted through the circulatory system, during the suspension of respiration, would seem to act like a narcotic poison upon the brain; no sooner therefore does it enter that organ, but deleterious effects are produced, the animal at first falls into a state of stupor, the pupils of the eyes become dilated, the respiration laborious, the muscles of the body convulsed, and the animal dies, *poisoned by its own blood.*" 37.

To be sure these extracts, as usual, bear very little on legal medicine; but they are more appropriate in a miscellaneous medical journal than in a work professedly on Medical Jurisprudence. The following are more in character.

The body of a person who has died from drowning exhibits a physiognomy which it is proper to notice. The whole surface is distinguished by a remarkable coldness and pallor—the eyes are half open, and the pupils much dilated—the tongue is pushed forward to the internal edges of the lips and sometimes wounded—the mouth and nostrils are covered with foam. Sometimes, instead of a pallid visage, we have it swelled, and bloated with livid blood. On dissection, the vessels of the brain are more or less gorged with blood—in the trachea a watery and bloody froth will be found—the lungs expanded, full of frothy mucus, and generally livid. "The right cavities of the heart gorged with blood, the left nearly empty." The stomach will generally be found to contain some water. The presence of water in the stomach and lungs has given rise to great controversy; for as it was found that no water entered these organs when a corpse was immersed in water, it was inferred that the presence of that fluid in these organs necessarily proved that the individual must have been plunged into the water during life. As a general proposition this may be admitted as correct, although there are exceptions with which the medical jurist ought to be acquainted.

"We may, for instance, suppose a case, in which the submerged person may be so plunged at once under water, as to have been suffocated without his previously coming to the surface, and when *as-*

*phyxia* has taken place, the powers of deglutition, on which the presence of water in the stomach wholly depends, are at an end; or we may suppose that the party in question faints from terror; a remarkable instance of this kind is quoted by *Foderè*,* from *Plater*, of a young woman, who having been condemned to be drowned for infanticide, fainted at the moment she was plunged in the water, and having remained for a quarter of an hour under its surface, recovered after being drawn out."† 39.

In respect to the bronchiæ and lungs the quantity of water forced into these parts during the dying struggles of a drowning man is extremely small. Mr. Brodie found less than a drachm in the bronchial vessels of a cat. But although the presence of frothy fluid in the lungs may be considered as presumptive proof that the person found in water had perished by drowning, the converse of this proposition is by no means established by the absence of such an indication.

The buoyancy of the human body is another point which has occasioned much discussion among our continental brethren in particular. It is not, however, worth discussing in this place. The human body being somewhat weightier than its bulk of water, sinks therein. About the fifth day the generation of gas in the abdomen raises the corpse to the surface, the head and legs downwards, the loins upwards. This, we believe with our authors, is generally the position; but we have seen bodies floating *belly upwards*, when the abdomen happened to be *greatly* distended with air. It has been said, that a different position from that described (loins uppermost) will take place where the person has been strangled before he is thrown into the water; it being contended that in this latter case the lungs will be distended with air, and consequently that the buoyant power will be in the thorax. This distinction we are disposed, with our authors, to reject. "The fact is, that, in relation to gaseous contents, the lungs are the same in strangled as in drowned persons; for in both cases a quantity of air is forcibly expelled from them before dissolution."

*Hanging.* Although we must allow that the immediate cause of death in these cases is suffocation, yet it cannot be denied that other injuries are sustained, as pressure on the vessels and nerves, fracture of the spine, or dislocation of the odontoid process. It was long believed that apoplexy was

---

* "Medicine Légale, vol. iii. p. 85."

† "During such a state of the body there would be but a feeble call for oxygen; it is muscular action which so rapidly expends this important principle."

the cause of death in hanging; but it is proved that the pressure on the vessels of the neck only *occasionally* produces that sanguineous congestion in the brain which is remarked in apoplexy. Dr. Hooper has a preparation shewing this phenomenon. Gregory and Brodie have made experiments to shew that it is suffocation not cerebral congestion which is the usual cause of death. An experiment of Mr. Brodie's renders it probable that the pressure sustained by the nerves would occasionally cause death or much inconvenience, allowing that the person recovered from the other consequences of hanging. There can be no doubt that fracture or dislocation of the cervical vertebræ sometimes occurs, especially where the person is heavy or the drop high. Hanging is said by our authors (how can they tell?) not to be a painful death. The violent convulsions that follow the repeated and ineffectual struggles for breath "arise in consequence of the *dark-coloured blood* having reached the brain and spinal marrow, and the animal at this period is necessarily insensible." We apprehend there is much gratuitous physiology here. Have our authors never seen living and sensible bodies agitated by painful convulsive spasms? If they have not, others have. We have at least as good ground to believe that the first convulsive movements of a hanging man are accompanied by painful feelings, as that the black blood has got to the brain and rendered him insensible. But independently of this consideration, who can believe that the violent and repeated, but ineffectual efforts to breathe, are not accompanied by sufferings, however transitory, that no tongue can describe! The dramatic symptomatology of hanging, in the work before us, is supplied by Shakspeare's *Henry* VI. *Part* II. *Act* III. *Scene* 2, to which we refer our readers.

The post mortem appearances in the brain are the same as in drowning, but the lungs, of course, do not contain water. We do not perceive that our authors have been able to lay down any distinctive marks whereby we can discriminate the effects of common hanging from those of manual strangulation.

*Want of Oxygen.* We all know that if atmospheric air be deprived of oyxgen it is no longer capable of supporting respiration. Thus death takes place from exposure to the fumes of charcoal, lime kilns, cellars, caverns, wells, and dungeons. The history of the black-hole at Calcutta was too good to escape our authors, and accordingly *five pages* are occupied with the details! The connexion between this diabolical piece of eastern tyranny and medical jurisprudence, we are totally unable to perceive, and its physiological bearings might have been stated in less than half a page. But we are sorry to say that the work before us appears to be constructed on the same principles as the French *Dict. des Sciences Medicales.* The authors of both productions appear to have entertained a horror of the word "*finis,*" like that which we feel towards annihilation; and both seem to have grasped at every collateral subject and circumstance to keep at a distance the dreaded determination.

The physiological remark elicited from our authors by Mr. Howell's melancholy narrative is this—that when oxygen is deficient we should keep ourselves as quiet as possible, since muscular exertion causes a greater expenditure, and consequently a greater demand for that constituent of respirable air.

There are other ways by which suffocation may be produced, as where the respiratory muscles are paralyzed, or have not room to act:—thus a person buried in a heap of ruins, though his head be free, may perish from the pressure of the surrounding substances; and in this way it is probable that criminals have died under the pressure made on their breasts by weights when they refused to answer questions, &c. Although the law which warranted this cruel process is obsolete or repealed, and consequently the practice abolished, yet the process is carefully detailed by our authors, agreeable to the general tenor of the work, which, in fact, treats "*de omnibus rebus et quibusdam aliis.*"

We shall not follow our authors in their serious refutation of the "incredible mode of suicide" said to be practised by slaves and negroes, namely, "swallowing their tongues"—nor the "voluntary suspension of breathing," by which some have been said to make their exit. The last mode of suffocation adverted to in this work is, from the entrance of foreign bodies into the aperture of the glottis. Instances of this kind are so familiar to every body that, were it not for learning's sake, it might appear unnecessary to record the deaths of Anacreon and Gilbert, of poetical celebrity, the former of whom perished by a grape seed, and the latter by a piece of mutton. A curious case has been communicated by Mr. Alcock, of a patient who died in an epileptic fit with which he was seized after a hearty meal of pork. On opening the trachea it was found to contain a quantity of animal matter resembling the meat he had eaten.

*Exposure to Cold.* The histories of Sir Joseph Bankes, the Northern Expedition, the Russian Campaign, &c. are all familiar illustrations of the effects of a low temperature on the human frame.

" *Animal heat*, as Mr. *Brodie* observes, *is in some way or other dependant upon the integrity of the functions of the Nervous System;* and consequently the absolute degree of cold which an animal can bear with impunity will, *cæteris paribus*, be determined by his powers of producing heat; we must therefore cease to regard the fact as extraordinary, that an animal, which is under the influence of a deleterious narcotic poison, or in whom, from any other morbid cause, the powers of the nervous system are exhausted, may be destroyed by a diminished temperature, that would scarcely affect even the sensations of one, differently placed in relation to his nervous energy; thus it is with a person in the last stage of intoxication, in whom the powers of life are ebbing, in consequence of the previous state of morbid excitement; in the course of the last winter, two instances occurred of drunken persons being taken to the watch-house: where, there not being any charge against them, they were dismissed by the constable of the night, and perished in the streets." 60.

Mr. Brodie classes the effects of cold in the following order:

" 1. It lessens the irritability, and impairs the functions of the whole nervous system.
" 2. It impairs the contractile powers of the muscles.
" 3. It causes contraction of the capillaries, and thus lessens the superficial circulation, and stops the cutaneous secretion.
" 4. It probably destroys the principle of vitality, equally in every part, and does not exclusively disturb the functions of any particular organ." 61.

*Death by Lightning.* Thunder and lightning are unquestionably electrical phenomena. The human body is alike affected by both. The experiments of Mr. Brodie would lead us to suppose that the lightning or electric shock does not destroy vitality equally in all parts of the body, as was believed by John Hunter and others, but that, on the contrary, the functions of the brain were those on which the electric shock exercised its primary influence. We shall not follow our authors in their elementary disquisitions on the nature of thunder and lightning, and whether life is always lost by the discharge from the cloud to the earth, or from the earth to the cloud. All these things are taught in the chemical lectures and chemical writings familiar to the profession. It is perfectly evident that if such elementary matter necessarily compose a part of medical jurisprudence, then there is no branch of science unconnected therewith—consequently the whole life of man—at least of a medical man, may fairly be devoted to medico-legal researches.

*Starvation.* The power of abstinence from food varies with the age of the individual. He will perish from inani tion with a rapidity proportioned to his youth and state of robust vigour Women are able to support abstinence longer than men.

"It has been also observed that a moist atmosphere contributes to the protraction of life, under circumstances of privation; this may depend, not only upon the fluid matter thus furnished to the body, but upon the non-conducting power of the medium, in relation to aqueous vapour: the ingestion of a very small proportion of water revives in an extraordinary degree, the animal perishing from famine, and prolongs his existence. *Redi* instituted a series of experiments with the sole view of ascertaining how long animals can live without food. Of a number of capons which he kept without either solid or liquid food, not one survived the ninth day; but one to which he allowed water, drank it with avidity, and did not perish until the twentieth day. *Elizabeth Woodcock*, who was buried under the snow, near Cambridge, for the space of eight days, undoubtedly owed her preservation to the snow which she occasionally sucked."* 68.

The extraordinary cases of fasting recorded in various transactions and journals are justly considered as impositions on the credulity of mankind. Our authors detail in four pages the history of Viterbi's voluntary starvation in one of the prisons of Corsica, as recorded in the Medical and Physical Journal for March 1822. He lived nearly three weeks before he effected his dreadful purpose. A much more curious case is related in Falret's. The siege of Jerusalem, and the shipwreck of the Medusa, with the dreadful raft scene, close this section of the work.

*Treatment of Asphyxia.* This is an important subject, and we were greatly surprised to find that the ideas of our authors coincided with our own upon some essential points wherein we have long differed from the general tone of professional as well as popular tenets. From circumstances unnecessary to mention here, we have had numerous opportunities of seeing and also of practising the usual means resorted to for restoring suspended animation, especially in cases of drowning; and we are sorry to say our experience and observations by no means tended to confirm the gratifying reports which we every day see made by humane societies and their now numerous affiliations. Our attention, therefore, was strongly excited by the following passage which opens this section.

---

* "This event occurred during the period of the author's studies at Cambridge; and he can therefore offer his testimony to the truth of the statement; he visited the woman soon after her disinterment." 68.

"But the fact is not to be concealed, that the medical profession, as well as the public, have long been too sanguine in their estimate of the probabilities of recovery by art, in cases where life is suddenly arrested by the operation of external causes; and upon this occasion, the establishment of the 'ROYAL HUMANE SOCIETY for the recovery of persons apparently dead,' requires some notice, in relation to the possible extent of its successful exertions. Without some explanation it will be impossible to reconcile the reports of that philanthropic institution, with the physiological views which we have attempted to establish in the present work; it therefore becomes a part of our duty to explain the nature of the fallacies into which the witnesses and reporters of cases of suspended animation appear to us to have been unconsciously betrayed, and which have so frequently bestowed upon fable the colour of truth, and given to vague report, the apparent stability of credible testimony. In the first place we would observe, that in those cases in which a long interval is stated to have occurred between the suspension of breathing, from drowning, and the restoration of that function by art, it is probable that the anxiety of by-standers who witnessed the struggles, and the impossibility of justly appreciating the lapse of time in such moments of anxiety* and distress, have led to the erroneous statements with which the subject is embarrassed. There is, moreover, another fallacy into which the anxious observer is very likely to fall,—the sufferer may have breathed unobserved during the alleged interval of asphyxia; and if this fact be admitted, we at once reduce some of the most incredible of these reports to the rational standard of physiological probability. Nor shall we hesitate in the present chapter to offer our remarks upon the plan of recovery proposed by this society with as much freedom, and as little reserve, as we have ventured to question the literal accuracy of their reports. But while, thus fortified by physiological arguments, we profess to discredit many of the results stated by this society, let it not be supposed that we would prefer a charge of insincerity against their authors, or attempt to withhold any portion of that public patronage and consideration, to which their zeal and philanthropy so justly entitle them." 77.

The following may be considered as the principal resources

* "That which we call duration is in fact a feeling of succession, and is computed by the number of ideas that pass through the mind; whenever an event occurs which powerfully excites the attention of an observer, he watches the most minute change, whence he believes that the time which elapses before the whole event is completed, appears to be unusually prolonged. When the infidel sultan of Egypt refused to believe that Mahomet could have ascended into the seven heavens, and have held some thousand conferences with the Almighty in the space of a few minutes, the learned mussulman, who was consulted on the occasion, endeavoured to turn His Majesty to a more strict faith, by demonstrating to him that a short period of time became converted into a long one, when a great multitude of important events were crowded into it."

upon which the Humane Society rely for the restoration of suspended animation, viz. inflation of the lungs—application of heat—internal exhibition of stimulants—friction—electricity—cool air—blood-letting.

From Mr. Brodie's manuscript notes much information is derived by our authors on the first or most important measure, the inflation of the lungs. Mr. B. considers a common pair of bellows preferable to those manufactured for the Humane Society. Single ones are sufficient, for the air will escape from the lungs without suction. It is not advisable to insert the nozzle in a wound in the trachea. A tube may be introduced into the rima glottidis, which can be effected without much difficulty; but Mr. B. thinks it more expedient to inflate the lungs by means of a tube inserted into one nostril, keeping the other and the mouth carefully closed. The bellows thus disposed, the air should be driven into the lungs with a certain degree of force. They will thus become inflated, and in the intervals of inflation the air will escape by the mouth and the other nostril. There is one objection, however, to this process—the escape, at each inflation, of a portion of air into the stomach—an event, if possible, to be avoided, since distention of the stomach will prevent the descent of the diaphragm, and thus prevent a perfect inspiration. To obviate this, the thyroid cartilage should be pressed close back upon the upper part of the œsophagus, so as to close it against the ingress of air. All the operator has to do is to inflate the lungs—the deflation will be produced by the elasticity of the ribs, and the pressure of the abdominal muscles and viscera. As for the inflation by oxygen gas—its propriety is doubtful, and loss of time is certain.

*Application of Heat.* The bath is preferred by our authors to all other means; but they seem to forget that in not one case of 500 can this process be employed without fatal delay. Warm flannels, bags of sand, or any thing that comes first to hand, should be the means employed.

*Galvanism.* The sanguine expectations of physiological enthusiasts, respecting this means of resuscitation, have ended in disappointment.

"We are bound to confess that although *galvanism is capable of exciting extraordinary contractions in the* VOLUNTARY *muscles, and of astonishing the multitude, yet its influence does not extend to those that are* INVOLUNTARY. *Bichat* states distinctly that *the involuntary muscles are beyond the reach of galvanism.* Mr. *Brodie* has frequently attempted to restore the heart's action by the galvanic stimulus, in an animal dead from syncope, but never with success." 83.

The bellows are preferable in all respects to the galvanic trough.

*Particular Cases of Asphyxia.* Where the action of the heart fails before that of the respiratory organs, artificial inflation can be of no service. In syncope, our authors think that the introduction of wine, volatile alkali, and other stimulants into the stomach may be serviceable—but not so in suffocation.

*Cessation of Respiration, the Heart continuing to circulate Black Blood.*

"It has been stated that in cases of suffocation the heart continues to contract for a short period, after the cessation of breathing; that this interval is extremely short, but liable to vary from several causes; and that it is uniformly shorter in cases of death by drowning than in those by strangulation. To the physician this is an interval of anxiety and importance; let him beware how he trifles with the fleeting moments, in which alone the resources of his art can be of any avail. If artificial respiration be established at this period, the blood will become once more oxygenized, the action of the heart will be continued, the scarlet blood will be transmitted to the brain, and sensibility will therefore return; the nervous energy will be once more transmitted to the respiratory organs, and the animal will at length make a voluntary effort to inspire air. Here then is the interval of time, during which artificial breathing may be employed so as to effect a restoration to life, where death must otherwise have been inevitable. Mr. *Brodie* has made a great variety of interesting experiments upon this subject, from which may be deduced the following important corollaries.

"1. If the lungs be inflated, the action of the heart will continue.

"2. If the action of the heart has become feeble, but the circulation is nevertheless not entirely suspended, the inflation of the lungs will cause the feeble actions to become again frequent and vigorous.

"3. If the action of the heart has entirely ceased, it is impossible to restore it by the inflation of the lungs.

"4. If the action of the heart has not entirely ceased, but is so feeble as no longer to maintain the circulation, the artificial respiration will prove as useless, as if the heart were perfectly motionless." 87.

As it is stated by our authors that the heart rarely if ever continues to act more than four or five minutes after respiration ceases, it follows that this is the only interval in which there is any chance of renewing the suspended animation! If this be the case, (and we fear it is too near the truth,) what becomes of all those boasted re-animations after *hours* of asphyxia?

It has been proposed, in cases of suffocation, to abstract blood from some of the larger veins. This measure, our authors think, can be of no use as far as relates to the asphyxia; "but incidental benefit may arise where congestion has taken place in the brain, as happens in hanging." "Cordials, moderate warmth, and quiet, are the resources upon which we are to rely for the ultimate recovery of the vital powers, after the complete establishment of the function of respiration." The injection of tobacco, in cases of asphyxia, a practice which, until within a few years, was annually recommended by those "who professed to instruct the profession," is considered by our authors "*as one of the most stupendous errors that ever occurred in the exercise of the healing art.*" We cannot but look on this passage as breaking a fly on a wheel; for according to the physiological principles laid down in this work, which we fear are too correct, ninety-nine in the hundred of those resuscitations which we read of must have been *fabulous*, and therefore it is highly improbable that tobacco glysters have a single life to answer for. Can this be said of some other errors in the healing art —for instance, the healing regimen in eruptive diseases, and the Brunonian treatment of fevers? To these, rather than to fumes or infusions of tobacco, the epithet "stupendous" might more justly be applied!

Where the asphyxia has arisen from the inhalation of noxious vapours, as those of charcoal, exposure of the body to cold is recommended, in addition to the common means. It is hardly necessary to allude to the antiquated and erroneous notion of water getting into the lungs, and thus being the cause of drowning; nevertheless it appears that a medical practitioner lately acted on this principle, and hung up his patient by the heels! This not succeeding, he gave his opinion in court that the man could not be recovered in consequence of the *impurity of the water arising from the gas works!*

We need not rake up the many stories of men recovering after being hanged, and of having their throats cut previously to being turned off, in order to save their lives while suspended. It is certain that some have escaped the gallows in this way; and no instance, we believe, is better authenticated than that of Margaret Dickson, of Musselburgh, who was tried, convicted, and hanged, for the murder of her own child. After execution, her body was cut down and delivered to her friends for interment. While they were transporting it in a coffin to her native place, she popped out her head, and the bearers took to their heels. A person, however, from a public house went to her assistance, and conveyed her to bed. Next morning she walked home, and lived twenty-five years afterwards!

*Coroner's Inquest.* This short section is legal; but contains some judicious reflections on the office of coroner.

*Suicide.* Our authors are not disposed to look upon suicide as always an act of insanity. It may be a matter of sober calculation between two evils, though, we imagine, this can rarely be the case. In nine instances out of ten we believe there is an hallucination of the judgment, or, in other words, mental derangement on some point or points connected with the act of self-destruction. As to the punishment which human laws inflict, they can only act on what the criminal has left behind him—his reputation and fortune; on the former, by indignity to the corpse, (now done away)—on the latter, by forfeiture of property. Our authors do not attempt the solution of the question, whether excessive punishment does or does not defeat its object; but they properly suggest that coroners should be more strict in having the bodies carefully examined anatomically.

"We will not maintain with a French author on Medical Jurisprudence, that the signs of insanity can often be discovered on dissection; though we can imagine some cases, as where there has been an excessive determination of blood to the brain, in which this inspection may be satisfactory; (See vol. 1, p. 327.) *Fourcroy* and *Durande* have also found, on dissecting persons who had committed suicide, hardness of the liver, and gall stones; and *Foderé* observes that, in failure of other evidence, such appearances deserve to carry some weight. But benefit would still result from the practice; first from the general horror in which dissection is held, for if the dread of an ignominious burial, however remote the chance of its infliction, can be supposed to discourage this offence, under the existing law, the certainty of personal mutilation would operate in the proposed alteration." 104.

*Murder; and first by wounds.* These are divided by our authors into incised, punctured, lacerated, and gun-shot wounds, on each of which some observations are made. They properly remark that no graduated scale of wounds, expressive of the degree of danger or curability, can ever be formed. The constitution, temperament, age, habits of life, &c. must be taken into account as well as the situation and extent of the wound. The surgical practitioner, therefore, will be cautious in his prognosis, when he recollects the many instances on record of wounded brain, lungs, and abdominal viscera, recovering. In examining wounds the surgeon should endeavour to disturb as little as possible, at first, the position in which the body is found. The scalpel must afterwards investigate its particular conditions and relations. But the general directions of our authors are too vague to enable us to cull out any specific information for these pages.

*Poisons.* Toxicology forms one of the most important and elaborate branches of forensic medicine—a branch that has been enlightened, within the last few years, by many valuable discoveries of chemistry and physiology. That engines so powerful and secret as poisons should have universally excited the terror of mankind is a fact that cannot surprise us, and when we consider how intimate are the relations between fear and credulity, we need not seek farther solution of the many problems to which the exaggerated statements of the ancient toxicologists have given origin. Plutarch, Tacitus, Livy, and others, are full of wonderful stories respecting poisons; nor are modern records unfruitful of the same. The celebrated Tophena, of Palermo, may be considered as the locust of modern history—the drops which she invented being sold under the appellatives of Aqua Toffania, Acquetta di Nipoli, &c.

"With respect to the secret modes in which poisons have been supposed capable of acting, makind have ever betrayed the most extravagant credulity, of which the numerous tales upon record afford ample proof; such as that reported of *Parasapis* by *Plutarch*, from *Ctesias*, in his life of *Artaxerxes*, who, it is said, by anointing a knife on one side by poison, and therewith dividing a bird, poisoned *Statira* with one half, and with the other regaled herself, in perfect security. We are also told of *Livia* who poisoned the figs on a tree which her husband was in the habit of gathering with his own hands. *Tissot* informs us that *John*, king of Castille, was poisoned by a pair of boots prepared by a Turk; *Henry* VI. by gloves;* Pope *Clement* VII. by the fumes of a taper; and our king *John*, in a wassail bowl, contaminated by matter extracted from a living toad. To these few instances of credulity may be added the offer of the priest to destroy queen *Elizabeth* by poisoning her saddle, and the *Earl of Essex*, by anointing his chair." 137.

The power of so graduating the force of a poison as to enable it to operate at any given period, seems to have been entertained by the earlier members of the Royal Society; but is now discarded—unless we take into account the slow *secondary effects* of a poison administered, whereby the constitution may be worn out.

---

* "The belief in the possibility of poisoning by the vestments is very ancient, as is shewn by the fabled death of Hercules."

——————— " 'Capit inscius heros:

Induiturque humeris Lernææ virus Echidnæ.

———————

———————

Incaluit vis illa mali; resolutaque flammis;

Herculeos abiit late diffusa per artus.' "

" *Ovid. Metam. Lib.* ix. *v.* 157."

*Slow Poisons.* There are several examples on record where substances have lodged in the alimentary canal for a considerable time without apparent injury, and afterwards proved fatal, as nuts, plumb stones, copper half-pence, &c. but how far such things as these could have been used intentionally, is very doubtful. It has also been supposed, and indeed asserted, that certain bodies, as powdered glass, enamel, diamonds, &c. slowly lacerate the coat of the stomach, and thus destroy life. Upon the same principle it is affirmed that human hair chopped fine, constitutes the active ingredient of a slow poison frequently employed in Turkey, for the purpose of producing a chronic disease resembling cancer in the stomach. Some have supported, others have denied these effects; but, as our authors justly observe, we may reconcile, in some measure, these clashing testimonies, by remembering that these mechanical substances may be a thousand times swallowed with impunity, while, under certain circumstances, the most trivial body may lodge in the intestines and produce death. The whole business of slow poisoning then may be resolved into the two following modes, viz. 1, consecutive poisoning—and 2, accumulative poisoning. The first is, where the patient, having recovered from the immediate effects of a single dose, suffers subsequently a series of symptoms from the injured structure to which it had given origin. The following case related by Orfila may serve as a specimen.

" *Maria Ladan* drank by mistake a spoonful of *aqua fortis*, the most violent symptoms supervened, but which by judicious treatment gradually subsided, when at length she passed by stool a long membranous substance, rolled up, and which represented the form of the œsophagus and stomach, and which, in fact, was found to be the interior membrane of these organs; from that moment the sensibility of the digestive organs became excessive, and two months after the accident she experienced a sudden shock and died." 147.

By the repeated administration of a substance in doses, of which no single one could occasion harm, a dangerous and even fatal accumulation in the system may ultimately take place. The administration of mercury is an illustration;—and perhaps arsenic, digitalis, and several other substances, might come under the same head.

*General Remarks on Medical Evidence in Cases of Poisoning.*

" The great constituents which form the medical proof of poisoning, are derived from Chemical, Anatomical, and Pathological researches: viz.—the existence of poison in the stomach or intestines; the morbid appearances, corresponding to such poison, upon dissection; and the characteristic symptoms which accompanied the action of it, previous to death. Where these circumstances occur in combination, the demonstration may be said to be complete, for we have arrived at absolute certainty." 153.

But scientific evidence, short of such perfection, may be sufficient to lead to conviction. The fact of a poison having been found in the body may supersede the necessity of pathological testimony; but pathological without chemical evidence will rarely be satisfactory. Here our authors endeavour to bring the more leading points of controversy within the scope of a few prominent questions, as follow :—

"Q. 1. *Whether all, or most of the symptoms, characteristic of the action of corrosive and narcotic poisons, may not arise from morbid causes of spontaneous origin?*

"Q. 2. *Whether organic lesions, similar to those produced by poisoning, may not occasionally result from natural causes?*

"Q. 3. *Whether the rapid progress of putrefaction, in the body generally, or in any particular part, is to be considered as affording any presumptive evidence, in favour of a suspicion of poisoning?*

"Q. 4. *How far the absence of poison, or the inability of the chemist to detect it, in the body, or in the fluids ejected from it, is to be considered as a negative to an accusation of poisoning?*

"Q. 5. *What degree of information can be derived from administering the contents of the stomach of a person supposed to have been poisoned, to dogs, or other inferior animals?*" 155.

*Question the first.* It must be admitted that the symptoms produced by *violent irritation* in the primæ viæ are not characterized by a diversity corresponding with that of the causes which may excite it. Thus the symptoms of cholera are induced without any ingesta of an acrid nature—and by poisons or strong emetic substances. Here the practitioner must be guided by various collateral circumstances, as the season of the year, the constitution of the patient, &c. Where corrosive poisons have been taken, there will generally be, in addition to the usual symptoms of cholera, a sanguineous vomiting, extreme burning in the œsophagus and region of the stomach, swollen countenance, great dryness and tumefaction of the fauces, fœtor of the breath, ischuria, with discharges of bloody urine, and ulcerations about the fundament. But it is to be remembered, that in certain circumstances the natural secretions of the liver and chylopoietic organs will occasionally become so acrid and depraved as to produce symptoms very much resembling poison, and not only symptoms but organic lesions. For this we have the authority of Galen, Aræteus, Morgagni, Hoffman, and many others, down to the late Dr. Curry. Morgagni's language on this subject is strong. "Facile agnosco à pravâ ipsâ corporis dispositione internum aliquando posse venenum gigni." Dr. Curry (not Currie, as our authors write the name) states his belief that, under a certain state of irritation, the biliary organs may secrete a bile of so very acrid a nature as to excite an almost immediately fatal impression upon the alimentary canal.

Q. 2. The organic lesions have created much doubt as well as the symptoms. Thus, among the signs of poison, disclosed by dissection, the separation of the villous coat of the stomach has been generally considered the most certain criterion. But every man who has been in the habit of much morbid dissection must have seen this phenomenon both in the stomach and intestines, where not the slightest ground of suspicion existed respecting poison. On the other hand, many vegetable poisons destroy life without causing any inflammation of the primæ viæ. But there remains another, and more redoubtable source of fallacy—the erosion of the stomach by the gastric juice.

" *Mr. John Hunter* details the history of three examples, in which the stomach was considerably perforated. Two of the men had died shortly after having their skulls fractured, and the third was a man who had been hanged, so that in each of these cases the person had been deprived of life by violence; whence *Dr. Adams** inferred, that *Mr. Hunter* limited the action of the gastric juice on the stomach to such as died from violent and sudden causes; and many physiologists have, accordingly, supposed that solution of the coats of the stomach never takes place, except where the person has died suddenly; this, however, is an inference, as *Mr. Burns* has very justly observed, 'by no means warranted by the general tenour of *Mr. Hunter's* essay,' indeed he expressly states, that 'there are few dead bodies in which the stomach is not, *at its great end*, in some degree digested;' 'and any one,' continues *Mr. Hunter*, 'who is acquainted with the art of dissection, can easily trace the gradations from the smallest to the greatest.' The consideration of the vast importance of this fact, and frequent opportunities of investigating the subject, induced *Mr. Burns* to collect the observations which he had made during the dissection of those bodies in which he found

---

* " *Adams's* Observations on Morbid Poisons, edit. 2, p. 30, where he says 'but for this purpose, *Mr. Hunter* saw that the animal must be in health immediately before death, otherwise neither the quantity nor quality of the secretion would be equal to the purpose; he was confirmed in this by the instances in which he saw the stomach digested; both were men who had died from a violent death; both had been previously in sufficient health to eat a hearty meal. The fair inference from these was, that when men die of disease, the appetite usually ceases, and probably the secretion of the gastric juice also."

the stomach digested; and these observations, he informs us, have led him to conclude, that the phenomenon in question is neither so rare in its occurrence as some have imagined, nor confined to such subjects as had been, previous to death, in a healthy condition; they have also convinced him, that other parts of the stomach, besides the large end, may be occasionally acted on by the gastric juice. 'That the digestion of the coats of the stomach after death is not a very rare occurrence, I think myself authorised to infer, from my having examined nine bodies in which the solution had proceeded to such an extent as to have made holes of considerable size through that viscus; and, besides these nine instances in which the digestion of part of the stomach was complete, I have had occasion to see, in opening this viscus, various degrees of dissolution of its villous coat."* 168.

In three of the instances alluded to by Mr. Burns, the patients had been worn out by debilitating diseases, being emaciated and anasarcous. That the solution of the stomach, in these, was correctly attributed to the gastric juice, is shewn by the following instructive dissection.

" 'I had occasion,' says *Mr. Burns*, 'two days after death, to open the body of a very emaciated and anasarcous young girl, who had died from scrofulous enlargement of the mesenteric glands. On raising the coverings of the abdomen, the stomach, which was empty, presented itself to view, *with its front dissolved*."† The aperture was of an oblong shape, about two inches in its long diameter, and an inch in its short, with tender, flocculent, and pulpy edges. This I demonstrated to the pupils attending my class; and I especially called their attention to the fact, that the liver, which was in contact with the hole, had no impression made on it. Having proceeded thus far, I placed all the parts as they had been, stitched up the abdomen, and laid the body aside in a cold situation for two days. Then I opened it again, in presence of the same gentlemen, and we found that, now, *the liver, where it lay over the dissolved part of the stomach, was pulpy; its peritoneal coat was completely dissolved, and its substance was tender to a considerable depth.* At this time the other parts of the liver were equally solid as before, and as yet every part of the subject was free from putrefaction; *the posterior face of the stomach, opposite to the hole, was dissolved, all except the peritoneal coat, at least the internal coats were rendered pulpy and glutinous; the*

---

* " 'It will generally be found that, where the coats of the stomach are softened by the gastric juice, the vessels are unable to resist the force of the syringe in injecting the body. In such subjects, therefore, we find the cavity of the stomach filled with wax, and we likewise see masses of it collected between the coats of the viscus.' "

† " Mark this circumstance, for we shall have occasion to revert to it, when we come to consider *the part* of the stomach which undergoes solution from the action of the gastric juice."

*peritoneal covering had become spongy and more transparent than it ought to have been.*' " 169.

These facts admonish the medical jurist that the appearances of these solutions will vary according to the period after death at which the body is examined. But the most satisfactory case which has been reported, in proof that the *post mortem* solution of the stomach may occur after a lingering disease, has been recently published by Dr. Haviland, in the Transactions of the Cambridge Philosophical Society, Vol. I. p. 287. The patient died of fever after an illness of 22 days; and, on opening the body 12 hours after death, the following appearances were noted.

" 'On raising the stomach and examining the little omentum, we were surprised by the appearance of a dark-coloured fluid, which seemed to escape from the former viscus. A most careful search was now made, and a large opening was perceived in the stomach on the upper and back part, near the cardia. The stomach was then detached, with a portion of the œsophagus and duodenum, when a large perforation of the diaphragm came into view, in the muscular part, corresponding precisely to, and communicating with, the hole in the stomach; so that a portion of the contents of the latter organ had escaped into the cavity of the chest. This part of the diaphragm was next removed. A careful examination of the other abdcminal and thoracic viscera did not lead to the detection of the slightest diseased appearance. There was no where the smallest evidence of previous inflammation, no adhesions or ulcerations of any part of the viscera. The fluid which had escaped appeared to be nothing more than the contents of the stomach, of which the wine and water* formed a part, and probably gave it its dark colour. The stomach, on being examined after its removal from the body, afforded the fol-following observations. The mucous membrane appeared to be more red and vascular than usual throughout its whole extent, and, here and there, were small spots of what seemed to be extravasated blood, lying below the mucous coat—for these spots were not to be washed off, nor to be removed by the edge of the scalpel. There were two holes in the stomach, the larger very near to the cardiac end of the small curvature, and on the posterior surface: this was more than an inch in length, and about half that breadth; the other not far from the former, also on the posterior surface, about the size of a sixpence. The edges of these holes were smooth, well defined, and slightly elevated. The coats of the stomach were thin in many other spots, and in one in particular nothing was left but the peritoneum, the mucous and muscular coats being entirely destroyed. The hole in the diaphragm was through the muscular portion, where it is

---

* "He had taken, at intervals, a small quantity of port wine and water."

of considerable thickness, and was large enough to admit the end of the finger. There was no appearance of ulceration or of pus adhering to the edges of this perforation of the diaphragm.' " 171.

Every man, indeed, who has been an attentive clinical observer must have witnessed many examples of the appetite reviving near the close of life, and continuing till the very last hour. In such cases the gastric juice must have its power, and may produce the phenomenon in question.

In respect to the *part* of the stomach acted on, Mr. John Hunter thought that the coats of this viscus would only be acted on at that part on which the contents of the stomach rested. In his practice the great end of the stomach (being the most depending part of the organ) was found to be chiefly affected—a fact which tended to corroborate his doctrine. But others having found the erosion in parts of the stomach which the gastric juice, *in the cavity of the organ*, could scarcely touch, the conclusion has been that—" we cannot ascribe the digestion of the stomach, in every case, to the gastric juice which has been *poured into the cavity* of that viscus—we are more properly, in some instances, to refer it to the action of the fluid *retained in the vessels* which *had secreted it.*" This is the creed of Burns and of the authors of the work under review. To us it appears that a fluid, say the bile, cannot be said to be *secreted* till it is in the very act of leaving the vessels which secreted it, and passing into the ducts or reservoirs destined to receive it. For example, the extreme vessels of the vena portæ change, or rather form the bile, while the blood is passing through them, and the secreted fluid is never such till it reach the pori biliarii. In *these* vessels *only* can it be " *retained*," but surely it will not be said that these are those " which had secreted it." Applying these observations to the stomach, the gastric solvent may be retained in the emunctories or conduits destined to convey it from the point at which it was formed or secreted to the inner surface of the organ, and there it may act in the manner described; but we maintain that it is incorrect physiology to say that a secreted fluid can be *retained* in the vessels " which *had* secreted it." It is, as we have observed, retained in the conduits leading from the secerning vessels.

" With respect to the appearances, which such erosions assume, some difference of opinion has also unfortunately existed. *Mr. Hunter* has asserted that 'there are very few dead bodies, in which the stomach is not, *at its great end*, in some degree digested; and the anatomist,' says he, ' who is acquainted with dissections can easily trace the gradations from the smallest to the greatest. To be sensible of this effect, nothing more is necessary than to compare the inner surface of the great end of the stomach, with any other part of the inner surface; what is sound will appear soft, spongy, and granulated, and without distinct blood-vessels, opaque, and thick, while the other will appear smooth, thin, and more transparent, and the vessels will be seen ramifying in its surface; and upon squeezing the blood which they contain, from the larger to the smaller branches, it will be found to pass out at the digested ends of the vessels, and appear like drops on the inner surface.' This condition, however, of the vessels does not invariably accompany such solution. In three of the subjects dissected by *Mr. Burns*, there was no appearance of vessels ramifying on the coats of the stomach." 173.

We must expect much difference of appearance in these erosions according to the state of the subjects before death, and therefore it is impossible to reconcile all the different accounts by one particular theory. Hunter characterizes them as having their edges apparently half dissolved, very much resembling that kind of dissolution which fleshy parts undergo when half digested in a living stomach.

" As certain corrosive poisons will occasionally produce such organic lesions in the stomach, as lead to perforations in its membranes, a question naturally arises, *how are we to distinguish such disorganizations, produced by causes acting during life, from those which result from solution after death?* To this we may at once return a general answer, that in a judicial investigation, we ought not to attribute erosion of the stomach to poison, except it be accompanied by evident marks of previous inflammation and reaction, or with gangrenous appearances; unless indeed the poisonous substance be found in the stomach, or the symptoms, previous to death, be characteristic and satisfactory." 176.

Here our authors have inserted a report of the memorable trial of Mr. Angus, of Liverpool, for the murder of Margaret Burns, his housekeeper, of which we need not take notice in this place, as it will be alluded to hereafter. The circumstances were very suspicious; but the ingenuity of the medical counsel (Dr. Carson) made it appear (whether true or false cannot now be known) that the erosion of the stomach was owing to the solvent power of the gastric juice; and that the appearances in the womb were equivocal. Better that ten culprits should escape than one innocent man be sacrificed.

" With respect to the possibility of confounding the appearances of gangrene, in the stomach, with those of putrefaction, some notice is necessary in this place; and we cannot better illustrate the subject, than by introducing the marks of discrimination which are considered by *Mahon* as decisive upon such occasions. The spots in the stomach, resulting from putrefaction, says he, may be distinguished from those which have resulted from violent causes, during life, in the following manner. If the stomach retain its natural colour, and the spots are mixed with a red hue, or the ulcers have pale, or bright red edges, such have been the effect of some violent impression upon the living membrane; whereas, on the contrary, if the stomach be pale, livid, or green, and exhibit spots of the same colour, but of rather a deeper hue, we may safely conclude that they are the genuine phenomena of putrefaction." 181.

Q. 3. The rapid progress of putrefaction after death is a popular argument in favour of poison having been swallowed. But this is very fallacious, as the putrefactive process is well known to take place very rapidly in people who have been cut off in the very prime of life and health. As far, however, as the observations of our authors extend, certain vegetable poisons appear to accelerate decomposition; for very shortly after death the body, under such circumstances, will frequently swell, become highly offensive, assume a black appearance, and exhibit gangrenous spots on its surface—an appearance alluded to in the following line of Juvenal:—

"Per famam et populum nigros efferre maritos."

This effect does not seem particularly to follow the mineral poisons. In the extraordinary case examined by Metzger, in which a very large quantity of arsenic had been taken, the body was not opened till eighteen days after death, and yet it exhibited neither fœtor nor black spots. Dr. Yelloly relates a case corroborative of the above. Morgagni and others, however, have given contrary evidence, and therefore the fact of accelerated or retarded putrefaction cannot be received with any confidence as a collateral indication of poisoning.

Q. 4. Of all proofs of poison none can be put in competition with the discovery of the substance itself in the stomach or its contents. The medical practitioner, therefore, must be prepared to ascertain, by every possible means, whether such substances are contained in the stomach; nor should any plea of putrefaction prevent the *post mortem* examination. The non-detection of poison, however, is no positive proof that none was administered; because the deleterious substance may have been absorbed or eleminated, during life—it may have undergone chemical changes, or entered into new combinations whereby its characters are masked or wholly changed. Many cases are on record illustrative of these remarks. Dr. Bostock has shewn that an animal may be killed by receiving a metallic poison into the stomach, and yet the most delicate chemical re-agents may not be able to detect any portion of it in the stomach after death. In a case related by Dr. Henry, an ounce of oxymuriate of mercury had been swallowed, and the fluid ejected from the stomach only twelve hours afterwards exhibited no trace of the poison. Our authors have given the details of the case of poisoning by Mr. Murray, related in the 71st number of our Edinburgh cotemporary and analyzed by us in our last volume, page 203. Although our authors remark on this case that the pathological and anatomical facts leave no doubt as to the poison, yet we adhere to the opinion then given, that *these* (there being no poison found) could not have been sufficient, had there been no external or collateral evidence.

Q. 5. Nothing very decisive is to be gained by administering the contents of the stomach to animals. If they be *forced* to swallow, some of it will get down into the lungs, and produce asphyxia—if mixed with their food, it may become neutralized or changed—and, after all, the contents of a morbid stomach might prove fatal to a small animal though no poison had been administered.

"In some instances such experiments may prove nothing, in others they may afford only equivocal results, but which may add something to the general weight of circumstantial evidence; while others, again, may furnish proofs so unquestionable, as to leave no doubt upon the subject; such was the case in the instance of *Michael Whiting*, who was convicted of administering corrosive sublimate to his brothers-in-law, when it appeared in evidence that a portion of the poisoned dumpling was given to a sow, who in consequence became sick, and remained ill for several days." 197.

With these general remarks on medical evidence in cases of poisoning, we must close this article, reserving for our next the classification and the particular history of each poison separately. We have now advanced into the very heart of our subject, and it now, of course, becomes both interesting and instructive, compared with what preceded.

---

