# Errata

**Published:** 1823-12-01

**Authors:** 


					624 line 17 for Healing read Heating.
634 line 11 (bottom) for Eliminated read Eliminated.

				

